# RNA-seq Profiling Reveals Defense Responses in a Tolerant Potato Cultivar to Stem Infection by *Pectobacterium carotovorum* ssp. *brasiliense*

**DOI:** 10.3389/fpls.2016.01905

**Published:** 2016-12-20

**Authors:** Stanford Kwenda, Tshepiso V. Motlolometsi, Paul R. J. Birch, Lucy N. Moleleki

**Affiliations:** ^1^Department of Microbiology and Plant Pathology, Forestry and Agricultural Biotechnology Institute, Genomics Research Institute, University of PretoriaPretoria, South Africa; ^2^Division of Plant Sciences, James Hutton Institute, College of Life Sciences, University of DundeeDundee, UK

**Keywords:** *Pectobacterium*, potato, plant defense, soft rot enterobacteria, *Solanum tuberosum*, RNA-seq, plant innate-immunity

## Abstract

*Pectobacterium carotovorum* subsp. *brasiliense* is a member of the soft rot Enterobacteriaceae (SRE) family that causes tuber soft rot and blackleg diseases of stems in potato plants. Currently, there are no effective chemical strategies for the control of members of the SRE. Thus, an understanding of the inducible defense responses in stems of potato plants is important, particularly during colonization of the vascular system. Here, time-course RNA-sequencing analysis was used to compare expressed genes between a susceptible potato cultivar (*Solanum tuberosum* cv Valor) and a tolerant cultivar (*S. tuberosum* cv BP1) at 0, 6, 12, 24, and 72 h post-inoculation with *P. c. brasiliense*. In total, we identified 6139 and 8214 differentially expressed genes (DEGs) in the tolerant and susceptible cultivars, compared to mock-inoculated controls, respectively. Key DEGs distinguishing between tolerance and susceptibility were associated with negative regulation of cell death and plant-type cell wall organization/biogenesis biological processes in the tolerant and susceptible cultivars, respectively. Among these were DEGs involved in signaling (mainly MAPK cascade and ethylene pathway), defense-related transcription regulation including WRKY transcription factors, and downstream secondary cell biosynthesis. Together, our results suggest that *S. tuberosum* cv BP1 likely employs quantitative defense response against *P. c. brasiliense*. Overall, our study provides the first transcriptome-wide insight into the molecular basis of tolerance and/or resistance of potato stems to SRE infection.

## Introduction

Potato ranks fourth, after rice (*Oryza sativa*), wheat (*Triticum* aestivum), and maize (*Zea* mays), as the most important human food crop worldwide (http://www.fao.org/faostat/en/#data/QC). However, cultivated potatoes, like many other plants, are exposed to diverse abiotic and biotic stresses. Some of the most important bacterial pathogens of potatoes belong to the soft rot enterobacteriaceae (SRE) consisting of *Dickeya* and *Pectobacterium* spp. In South Africa, *Pectobacterium carotovorum* subsp. *brasiliense* is the most widespread and aggressive soft rot enterobacterium, causing stem rot and blackleg in the field as well as tuber soft rot during post-harvest storage (van der Merwe et al., 2010). Incidentally, the global significance of *P. c. brasiliense* is growing with reports in countries such as Brazil, Canada, USA, New Zealand, China, and South Africa (Duarte et al., 2004; Glasner et al., 2008; van der Merwe et al., 2010; De Boer et al., 2012; Panda et al., 2012). Amongst the SRE are broad-host-range necrotrophic bacterial pathogens that employ plant cell wall degrading enzymes (PCWDEs) to macerate host tissues and obtain nutrients from dead cells (Davidsson et al., 2013). However, evidence suggests that soft rot bacteria can also exist as hemibiotrophs, living within the plant tissue (or in the surrounding environment) in an asymptomatic biotrophic state and only switching to a necrotrophic feeding mode when environmental conditions are favorable (Toth and Birch, 2005; Davidsson et al., 2013). The fact that the SRE localize deep inside the xylem or tuber lenticels makes effective control very difficult. Consequently, as with other vascular-dwelling pathogens, there are no efficient chemical control measures against SRE. Thus, the use of resistant cultivars remains the most desirable option of combating SRE (Charkowski, 2015).

Global gene expression studies of potato responses to environmental stresses such as drought, heat, and salinity (Massa et al., 2013; Gong et al., 2014) and biotic stresses as a result of fungal infections, predominantly caused by *Phytophthora infestans*, the causal agent of potato late blight, have been studied (Gyetvai et al., 2012; Gao et al., 2013; Massa et al., 2013). Currently, little is known about the molecular basis of potato resistance to soft rot phytopathogens, with only a few commercial cultivars exhibiting tolerance to challenge by these bacteria (Charkowski, 2015). Fortunately, the availability of the potato genome sequence and next generation sequencing approaches such as RNA-seq (Wang et al., 2009; Consortium, 2011), now make it possible to conduct in-depth transcriptome studies in deciphering the potato defense transcriptome in response to soft rot bacterial infection, particularly against the emerging phytopathogen, *P. c. brasiliense*.

Invasion of plants by microbes activates plant immune responses which limit proliferation of pathogens and arrest disease establishment. Plant immune responses are complex and vary depending on whether the invading pathogen is of biotrophic or necrotrophic lifestyle (Mengiste, 2012). Thus, plant immune responses are composed of pathogen-associated molecular pattern (PAMP)-triggered immunity (PTI) and effector-triggered immunity (ETI) pathways (Jones and Dangl, 2006). Generally PTI confers quantitative resistance in recognition of PAMPs (such as bacterial flagellin) and damage-associated molecular patterns (DAMPs) which mainly encompass degradation products from host cells due to the action of cell wall degrading enzymes. Accordingly, plant resistance to infection by broad host-range necrotrophs such as pectobacteria is quantitative, and it requires many genes to confer resistance. Perception of P/DAMPs by pattern recognition receptors (PRRs) on extracellular surfaces of plant cells in the apoplastic space leads to the induction of typical PTI responses such as ethylene/jasmonate hormone biosynthesis and cell wall modifications, resulting in inhibition of disease proliferation (Mengiste, 2012). However, when invading pathogens successfully suppress PTI, by injection of effector proteins directly into plant cells, ETI is activated, wherein, recognition of effectors in a gene-for-gene defense pathway leads to a hypersensitive response (HR) and cell death at infection sites resulting in disease resistance (Jones and Dangl, 2006). Induction of PTI or ETI activates mitogen activated protein kinases (MAPKs) for signal transduction and regulation of downstream pathogen responsive genes involved in plant resistance to pathogen attack (Zhang and Klessig, 2001). PTI and ETI signals converge in the MAPK cascade pathways and generally give rise to similar downstream responses. However, PTI is mostly effective against necrotrphic pathogens and PTI-related downstream responses include defense gene activation (Dodds and Rathjen, 2010; Meng and Zhang, 2013).

We previously reported on a potato cultivar *S. tuberosum* cv BP1 that shows significant tolerance to *P. c. brasilense* strain 1692 *(Pcb1692)* compared to the more susceptible *S. tuberosum* cv Valor (Kubheka et al., 2013). Thus, we wanted to use this tolerant vs susceptible model to further dissect the molecular basis of tolerance in *S. tuberosum* cv BP1, particularly in the early stages of infection (0–72 hpi) that signify the transition from asymptomatic to symptomatic phase in *Pcb1692* within the susceptible cultivar. Hence, in this study, we employed a time-course RNA-seq analyses to unravel the defense response in these potato cultivars during stem based colonization and infection by *Pcb1692*. The RNA-seq analysis allowed us to identify 6139 and 8214 DEGs in cultivars “BP1” and “Valor,” respectively, compared to mock-inoculated controls, in the time-course. Expression profiles of the differentially expressed genes and gene ontology enrichment analysis revealed that the MPK3/MPK6 cascade, WRKY33 transcription factor and downstream defense genes including secondary wall biosynthetic genes are probable key components in the potato defense responses to *P. c. brasiliense*.

## Materials and methods

### Plant material and RNA preparation

Seed tubers of *Solanum tuberosum* cv. Valor and *S. tuberosum* cv. BP1, susceptible and tolerant to *P. carotovorum* subsp *brasiliense* strain 1692 (*Pcb1692*) infection, respectively, were greenhouse grown under standard conditions (22 to 26°C, 16 h light/8 h dark photoperiod and 70% relative humidity). Stem inoculations were done following the approach previously described in (Kubheka et al., 2013), except that in this study we only used wild-type *P. carotovorum* subsp. *brasiliense* 1692 for the inoculations. Inoculated plants were assessed and sampled, within 2 cm above or below the point of inoculation, at 0, 6, 12, 24, and 72 h post inoculation (hpi) in triplicates (three plants were pooled together for each biological replicate). Samples at 0 hpi were mock-inoculated with MgSO_4_ buffer and used as controls. Total RNA was extracted from individual time-points and replicates independently using the QIAGEN RNeasy plant mini kit (Qiagen) including DNAse treatment (Qiagen). RNA was quantified using the NanoDrop (Thermo Scientific, Sugarland, TX, USA) and the quality and integrity checked using Agilent 2100 BioAnalyzer system (Agilent, Santa Clara, CA, USA).

### cDNA library construction and illumina sequencing

The construction of cDNA libraries and sequencing were carried out at the Beijing Genomics Institute (BGI-Shenzhen, China; http://www.genomics.cn/en/index). The quality of total RNA samples from individual biological replicates (*n* = 3) from each time-point was assessed using Agilent 2100 Bioanalyzer (Agilent RNA 6000 Nano Kit) and NanoDrop, and 200 ng aliquots were used for poly(A) mRNA isolation and preparation of cDNA libraries using the TruSeq RNA sample Prep Kit v2 (Illumina, San Diego, CA, USA) following manufacturer's instructions. The libraries were quality checked and quantified using Agilent BioAnalyzer 2100 system and qPCR. Finally, the libraries were sequenced with an Illumina HiSeq 2000 sequencer generating 90 bp paired-end reads. The data have been deposited in NCBI's Gene Expression Omnibus (GEO) and are accessible through the GEO accession number, GSE74871.

### Differential expression analysis and functional enrichment analysis

Paired-end reads from each time-point in each cultivar were initially quality checked using FASTQC (http://www.bioinformatics.bbsrc.ac.uk/projects/fastqc) and mapped to the potato reference genome using TopHat2 (version 2.0.13) (Trapnell et al., 2009). Transcript reconstruction was done using Cufflinks software tool (version 2.2.1) (Trapnell et al., 2012). HTSeq-count (Anders et al., 2015) and DESeq2 package (Love et al., 2014) were used to make read counts and perform a time-series differential expression analysis, respectively. A False Discovery Rate (FDR) threshold of 10% and an absolute log2 fold change > 1 were used to determine differentially expressed genes. Functional enrichment analysis of differentially expressed genes obtained from each comparison (direct pairwise comparison between cultivars “Valor” and “BP1” or cultivar specific comparisons of inoculated samples from each time-point to mock-inoculated controls) was performed using g:Profiler web server (Reimand et al., 2016). Orthology detection was performed using BLASTp searches to compare sequences of differentially expressed genes to the *Arabidopsis thaliana* TAIR genome using the ProteinOrtho software (Lechner et al., 2011), with the default cutoff E-value: 1.0E-05. Subsequently TAIR annotations were used in Figures and Tables in the text.

### RT-qPCR validation of RNA-seq data

For RT-qPCR, first-strand cDNA synthesis was done from total RNA using Superscript III First-Strand cDNA Synthesis SuperMix kit (Invitrogen, USA) following manufacturer's instructions. Quantitative real-time PCR using Applied Biosystems SYBR Green Master Mix was performed in the QuantStudio 12K Flex Real-Time PCR system (Life Technologies, Carlsbad, CA, USA). For RT-qPCR, 2 μl of sample was added to 8 μl of Applied Biosystems SYBR Green Master Mix and primers at a concentration of 0.4 μM. The cycling conditions were as follows: an initial denaturation at 50°C for 5 min and 95°C for 2 min followed by 45 cycles of 95°C for 15 s and 60°C for 1 min. Each sample was run in triplicate. The samples were normalized to 18S rRNA and elongation factor 1-α (PGSC0003DMG400020772, ef1α) as the reference genes and the mock treated samples used as calibrators (Nicot et al., 2005). The comparative CT (ΔΔ^ct^) method was used to measure relative expression (Livak and Schmittgen, 2001). Two tailed Student's *t*-test (unequal variances) was used to check whether RT-qPCR results were statistically different when comparing inoculated samples to mock-inoculated samples (^**^P < 0.01; ^*^P < 0.05). Primers used were designed online using Primer3Plus (http://primer3plus.com/cgi-bin/dev/primer3plus.cgi) and are listed in Table S9.

### Candidate novel CDS transcript validation

First-strand cDNA was synthesized as outlined in the RT-qPCR validation Section above. The PCR was done on Bio-RAD T100TM Thermal Cycler end-point PCR (Bio-RAD, USA). The PCR reaction mix consisted of 12.5 μl KAPA HiFi HotStart Ready mix (2X), 0.5 μM of each forward and reverse primer, 1 μl template cDNA in a final reaction volume of 25 μl. PCR conditions were: 98°C for 3 min; 28 cycles of 98°C for 30 s, annealing for 60 s, 72°C for 90 s, and final extension at 72°C for 5 min. The PCR products were analyzed on 1.5 % agarose gel including 1 kb DNA molecular weight ladder (NEB, UK). All the primers were synthesized by Inqaba Biotech, South Africa (Table S10).

## Results

### Illumina sequencing and reads assembly

Stems of two potato cultivars, *S. tuberosum* cv BP1 (tolerant cultivar) and *S. tuberosum* cv Valor (susceptible cultivar) were inoculated with *P. carotovorum* subsp. *brasiliense* strain 1692 (*Pcb1692*) and samples collected at 0 (mock-inoculated), 6, 12, 24, and 72 hpi. In total, 30 RNA samples (comprising three biological replicates) from stem tissues of these two potato cultivars were obtained from the five time points and subjected to RNA-seq. Approximately 1.4 billion paired-end reads were generated in the time-course, producing an average of 46 million mapped reads per sample (Table S1). In addition, over 80% of these reads could be mapped to the *S. tuberosum* reference sequence (*S. tuberosum* group Phureja DM1-3 516 R44), and approximately 92% were uniquely mapped (Table S1). The current potato genome has 38,982 predicted gene models (PGSC_DM_v4.03; http://solanaceae.plantbiology.msu.edu/pgsc_download.shtml) (Consortium, 2011). In this study, we identified expression of 38,688 genes by merging together transcripts reconstructed from each sample using Cufflinks software tool (v2.11) (Trapnell et al., 2012). Thus, the majority of annotated potato genes were detected (~ 99%). In addition, we identified 1828 candidate novel protein coding expressed loci, present in both cultivars (Table S2), based on the pipeline outlined in (Kwenda et al., 2016). These putative novel transcripts represent potentially new information for improvement of the current potato genome annotation.

### Transcriptional profiles in response to *Pcb1692* infection

**Figure 1:**
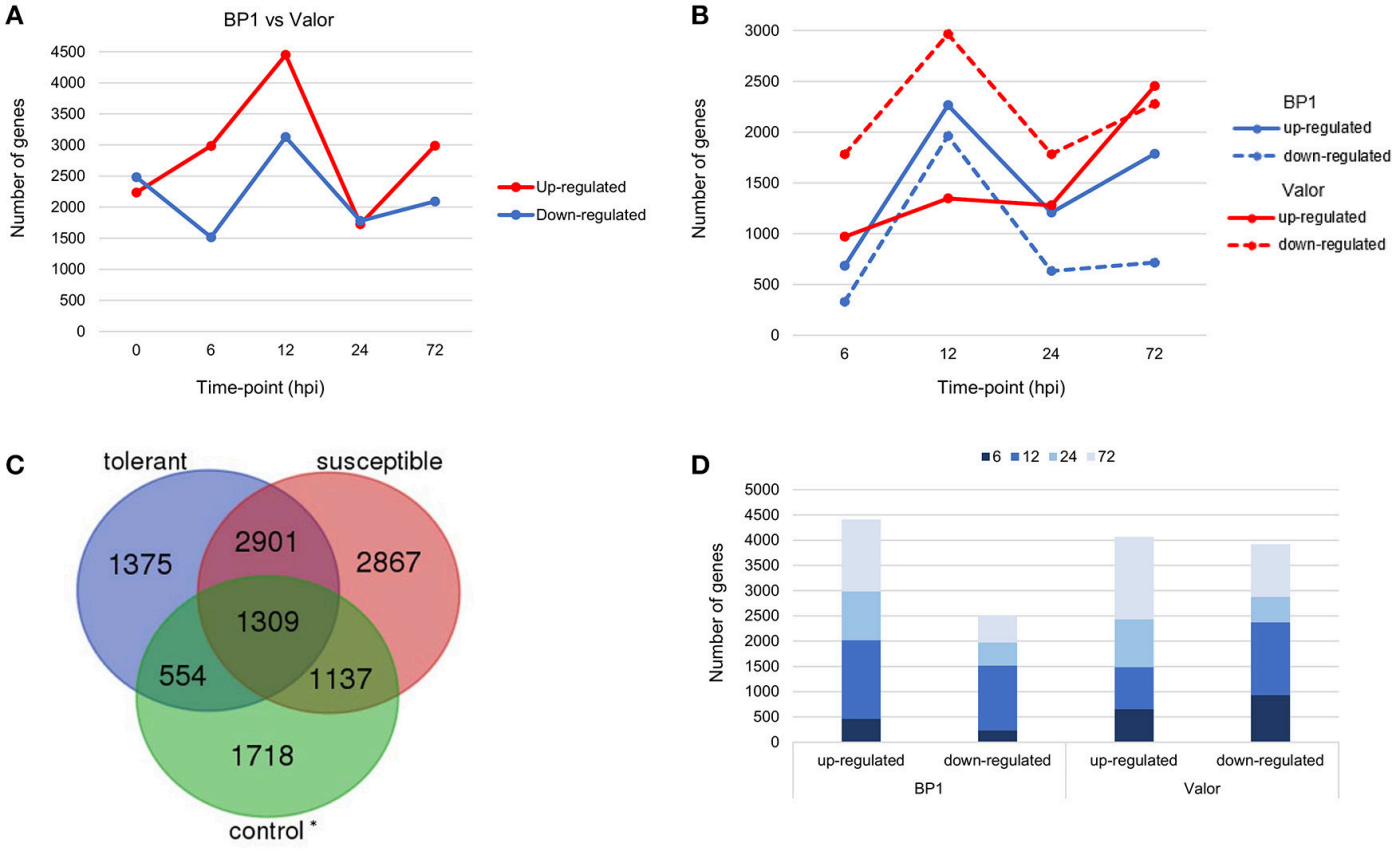
**(A)** Pairwise comparison of DEGs in cultivars “Valor” and “BP1” in the time-course showing number of DEGs up- and down-regulated in cultivar “BP1” compared to cultivar “Valor.” **(B)** Cultivar specific DEGs between inoculated samples and mock-inoculated controls in each cultivar independently. **(C)** In total, 1929 and 4004 DEGs were identified and are specific to the tolerant and susceptible cultivar, respectively. Of these, 554 and 1137 DEGs in “BP1” and “Valor,” respectively, represent intrinsic cultivar differences, and are related to plant growth and/or development (Table S5). In addition, 4210 DEGs were present in both cultivars in the time-course. ^*^Control group represents DEGs obtained between “BP1” and “Valor” at 0 h time-point. **(D)** Graph showing DEGs up- or down-regulated in both cultivars at individual sampling time-points (6, 12, 24, and 72 hpi).

**Figure 2:**
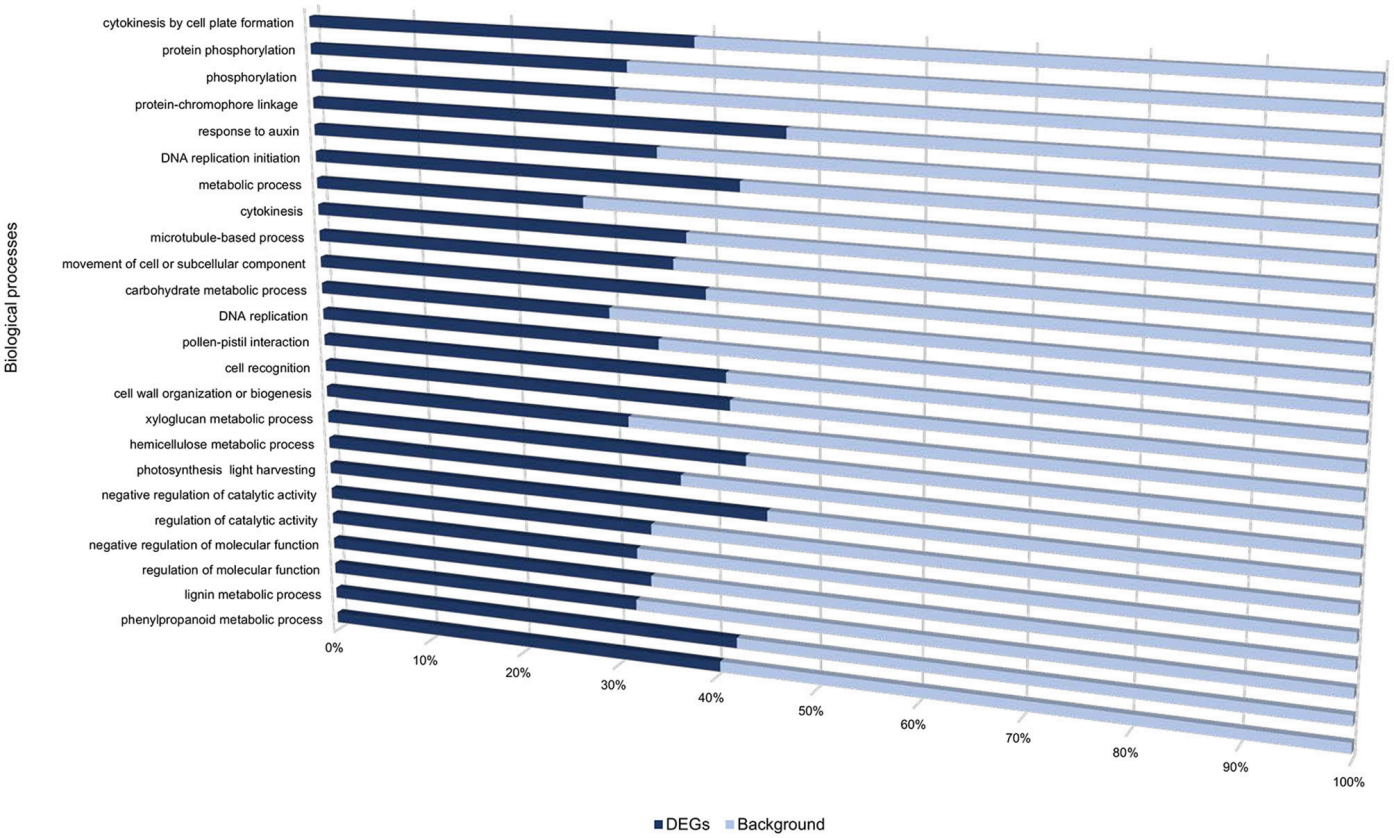
**Gene ontology biological processes overrepresented between cultivars “Valor” and “BP1” in the time-course**.

Transcriptome profiling revealed a total number of 4718, 4503, 7577, 3505, and 5081 differentially expressed genes (DEGs) between *S. tuberosum* cv BP1 and *S. tuberosum* cv Valor at 0, 6, 12, 24, and 72 hpi, respectively (Table S3). Transcriptional dynamics highlighting specific numbers of DEGs at individual time-points between these two cultivars, are shown in Figure 1A. These comparisons, over time, revealed an exponential increase of DEGs in the tolerant cultivar in the early hours of infection [0–12 hpi (Figure 1A)]. To investigate the functionality of the genes activated in response to *Pcb1692* infection in the tolerant cultivar compared to the susceptible cultivar, GO enrichment analysis was performed using g:Profiler web server (http://biit.cs.ut.ee/gprofiler/) against *S. tuberosum* ontologies (Reimand et al., 2016). Because of the large array of datasets over-represented under the three gene ontology categories namely; molecular function (MF), cellular component (CC) and biological process (BP), focus was only given to the BP responses in this study. The most overrepresented BP terms are shown in Figure 2.

To understand the transcriptional changes per cultivar in response to *Pcb1692* inoculation, cultivar-specific expression profiles were determined by comparing inoculated samples to their respective mock-inoculated samples (at 0 hpi). Toward this end, 6139 and 8214 DEGs were identified in the tolerant and susceptible cultivars, respectively (Figures 1B,C). We found that the number of DEGs was initially higher in the susceptible cultivar (2754 DEGs; 971 up-regulated and 1783 down-regulated) at 6 hpi, compared to only 1014 DEGs in the tolerant cultivar (684 up-regulated, 330 down-regulated) at this time point (Figure 1B). However, a marked increase in the number of DEGs in the tolerant cultivar was observed at 12 hpi (up to 2-fold increase in the number of DEGs compared to 6 hpi; Figure 1B). Furthermore, the highest number of DEGs was observed at 12 hpi in the tolerant cultivar (4227 total DEGs; 2265 up-regulated, 1962 down-regulated) (Figure 1B). Even though a peak in DEGs was observed at 12 hpi in the tolerant cultivar, the number of DEGs dropped significantly at 24 and 72 hpi. On the contrary, a marked increase of DEGs was observed at the later stages of infection (72 hpi) in the susceptible, and the highest number of DEGs was observed at this time-point (72hpi) (4732 total DEGs; 2455 up-regulated and 2277 down-regulated). Together, these differences in expression profiles between the tolerant and susceptible cultivars, indicate differences in defense responses in these two cultivars.

### The tolerant and susceptible cultivars employ similar sets of genes involved in pathogen recognition and wounding response

Among the identified DEGs, 4210 were present in both cultivars in the time course (Figures 1C,D and Table S4). Among these were membrane localized receptor like kinases (RLKs) including FLAGELLIN SENSING 2 (PGSC0003DMG400008296, FLS2), EF-Tu receptor (EFR, PGSC0003DMG400023283), Wall-associated kinases (e.g., WAK1) and Brassinosteroid insensitive 1-associated kinases (BAK1) (Table 1). FLS2 and EFR are key pattern recognition receptors (PRRs) that recognize the conserved bacterial flagellin and EF-Tu proteins, respectively, thus triggering PTI defense signaling pathways in plants (Gómez-Gómez and Boller, 2000; Boller and Felix, 2009). The large number of RLKs present in both cultivars, suggests that these two cultivars employ fundamentally similar sets of pathogen recognition genes (Table S4). Other DEGs related to plant defense responses were identified in both cultivars, including NAC domain-containing proteins and cytochrome P450 genes (e.g., PGSC0003DMG400030413) (Table S4). Furthermore, genes involved in pathogen perception and response to wounding were differentially expressed in both cultivars. These included signaling genes encoding transcription factors such as MYB, WRKY, AP2 (e.g., AP2-EREBP, PGSC0003DMG400002272) and ethylene response factors (e.g., PGSC0003DMG400041451, ERF1) (Table 1 and Table S4). These transcription factor families represent some of the major regulators of plant immune response pathways against necrotrophs (Lai and Mengiste, 2013). Additional wound responsive DEGs included RBOHD, lipoxygenases (e.g., LOX1, PGSC0003DMG400010859), NAC domain-containing proteins (e.g., NAC002, PGSC0003DMG400032555), *JAR1* (PGSC0003DMG400033879), and *JAZ10*, (PGSC0003DMG400006480) (Table 1).

**Table 1:** **Summary of selected pattern recognition receptors and intracellular receptors activated in response to *Pcb1692* infection**.

	**6 h^a^**		**12 h**		**24 h**		**72 h**			
**Potato gene ID**	**Valor**	**BP1**	**Valor**	**BP1**	**Valor**	**BP1**	**Valor**	**BP1**	**Arabidopsis ID**	**Gene name**
**PATHOGEN RECOGNITION**										
**RLKs**										
PGSC0003DMG400003195	6.47	4.54	–	12.46	–	3.34	6.49	2.35	AT3G05660	AtRLP33 (FLS2)
PGSC0003DMG400020697	–	–	6.59	2.58	–	–	3.25	7.06	AT5G46330	FLS2
PGSC0003DMG400020848	13.45	–	8.11	3.27	5.50	–	–	1.00	AT5G46330	FLS2
PGSC0003DMG400006502	–	3.32	3.18	5.82	2.66	6.06	11.88	5.58	AT3G47570	(EFR)
PGSC0003DMG400011932	–	–	4.03	–	–	–	3.32	2.69	AT3G47570	(EFR)
PGSC0003DMG400023283	5.58	6.11	–	44.32	7.31	9.71	6.77	12.21	AT5G20480	EFR
PGSC0003DMG400011792	0.21	5.54	–	15.78	–	11.79	–	10.20	AT1G21250	WAK1
PGSC0003DMG400025668	34.30	–	16.11	8.46	9.19	–	9.38	–	AT1G21240	WAK3
PGSC0003DMG400038918	8.17	–	–	4.47	–	–	–	–	AT2G23770	LYK4 (CERK1)
PGSC0003DMG401015527	19.48	6.38	–	11.92	6.80	4.61	7.79	–	AT2G19210	FRK1
PGSC0003DMG400003211	3.71	2.69	2.22	4.68	–	–	–	–	AT1G73080	PEPR1
PGSC0003DMG400020327	39.81	18.23	12.60	25.39	9.99	5.82	13.32	–	AT3G53810	LECRK42 (BAK1)
**NBS–LRR**											
PGSC0003DMG400001756	4.69	3.12	4.92	–	–	–	4.63	–	AT5G38344	
PGSC0003DMG400003353	8.57	33.82	–	–	–	62.25	37.27	76.11		
PGSC0003DMG400021469	4.03	–	–	–	2.93	2.71	3.39	2.33		
PGSC0003DMG400008185	0.45	–	0.52	0.34	–	0.44	–	–		
PGSC0003DMG400013627	0.04	–	0.41	0.41	–	–	–	–		
**TRANSCRIPTION FACTORS**											
**WRKY family**											
PGSC0003DMG402006935	–	–	–	614.38	–	480.44	38.10	1225.39	AT5G15130	WRKY72
PGSC0003DMG400021895	8.24	17.93	5.50	63.46	–	51.15	24.28	12.08	AT5G13080	WRKY75
PGSC0003DMG400009103	40.22	51.07	–	38.91	3.89	30.02	–	27.51	AT5G24110	WRKY30
PGSC0003DMG400016441	10.66	6.66	–	15.07	–	–	17.22	6.83	AT1G62300	WRKY6
PGSC0003DMG400018081	20.02	13.72	–	44.88	7.30	6.68	46.75	15.32	AT5G15130	WRKY72
PGSC0003DMG400019824	4.64	9.24	–	14.10	–	9.14	6.38	–	AT1G80840	WRKY40
PGSC0003DMG400020206	22.38	1283646.84	–	17915245.67	–	7114329.79	398.92	2487951.03	AT3G01970	WRKY45
**MYB family**											
PGSC0003DMG400001504	–	9.74	–	–	5.99	–	–	7.24	AT3G24310	MYB305, ATMYB71
PGSC0003DMG400003890	–	–	–	7.60	2.50	6.10	6.08	7.56	AT5G60890	ATMYB34, ATR1, MYB34
PGSC0003DMG400004612	1348.14	–	–	623.99	–	–	–	–	AT1G48000	MYB112
PGSC0003DMG400005641	5.11	–	–	4.00	–	–	–	–	AT2G47190	ATMYB2, MYB2
PGSC0003DMG400011048	32.93	3.32	–	13.06	25.02	19.40	59.43	43.77	AT3G09600	Homeodomain-like superfamily protein
PGSC0003DMG401010883	18.23	–	–	11.39	10.53	30.57	14.17	38.47	AT3G46130	ATMYB48
PGSC0003DMG402004611	–	–	–	9.35	–	8.15	4.17	–	AT2G47190	ATMYB2
**AP2/ERF family**											
PGSC0003DMG400002272	163.24	130.09	–	284.39	–	251.38	54.69	–	AT5G47220	ATERF2
PGSC0003DMG400016812	6.02	–	–	2.29	–	–	3.57	–	AT3G16770	RAP2,3, ATEBP, ERF72, EBP
PGSC0003DMG400026260	90.51	33.98	–	42.87	–	15.53	42.94	6.12	AT4G17500	ATERF-1, ERF-1
PGSC0003DMG400014594	3.85	–	2.67	4.41	–	4.22	7.02	–	AT3G23240	ERF1, ATERF1
PGSC0003DMG400026046	162813.36	1025615.53	13345.41	22905524.55	–	30550103.67	1762294.29	302026.73	AT2G44840	ATERF13, EREBP, ERF13
PGSC0003DMG400026261	105.16	73.89	–	83.78	–	17.26	52.96	–	AT5G47220	ATERF2, ATERF–2, ERF2
**Other wound-responsive genes**										
PGSC0003DMG400024754	24.62	9.59	294.80	19.98	7.55	1.00	6.48	–	AT1G09090	ATRBOHB, ATRBOHB-BETA, RBOHB
PGSC0003DMG400010859	18.86	29.73	–	226.35	–	85.36	8.07	17.27	AT1G55020.1	LOX1
PGSC0003DMG400006480	–	–	–	8.31	–	–	171.49	–	AT5G13220.1	JAZ10, TIFY9, JAS1
PGSC0003DMG400001223	8.37	9.47	106.28	15.45	7.62	10.81	3.46	5.46	AT2G43000	anac042, NAC042
PGSC0003DMG400039898	8.84	21.38	40.66	24.12	8.56	26.52	7.76	20.92	AT2G43000	anac042, NAC042

a *Fold changes in comparison to mock-inoculated controls*.

Interestingly, in addition to PTI related responses, several DEGs encoding R-proteins that predominantly contain a nucleotide-binding site (NBS) and/or leucine-rich repeat (LRR) domain, were differentially expressed in both cultivars at 12, 24, and 72 hpi (Table S4). Among these included resistance genes encoding R-proteins containing the coiled-coil (CC)-NBS-LRR and Toll interleukin 1 receptor (TIR)-NBS-LRR motifs (Table 1). Of these, PGSC0003DMG400001756 gene was up-regulated (~4-Fold) while PGSC0003DMG400008185 (CC-NBS-LRR) and PGSC0003DMG400013627 (TIR-NBS-LRR) were down-regulated compared to mock-inoculated samples of each cultivar (Table 1). Finally, different genes encoding CC-NBS-LRR, NBS-LRR, and CC-NBS-LRR motifs were differentially expressed at 72 hpi, with one *R*-protein encoding gene (PGSC0003DMG400003353) showing significant up-regulation (slightly over 32-Fold increase) in both cultivars (Table 1). *R*-gene mediated resistance leads to effector triggered immunity (ETI), a defense response which recognizes bacterial effector proteins. The actual role that the induced *R*-genes play in the two potato cultivars' response to *Pcb1692* is still unclear. Generally, ETI defense responses are not directly effective against necrotrophic pathogens such as *Pectobacterium* (Jones and Dangl, 2006).

### Cultivar-specific transcriptional changes following inoculation with *Pcb1692*

**Figure 3:**
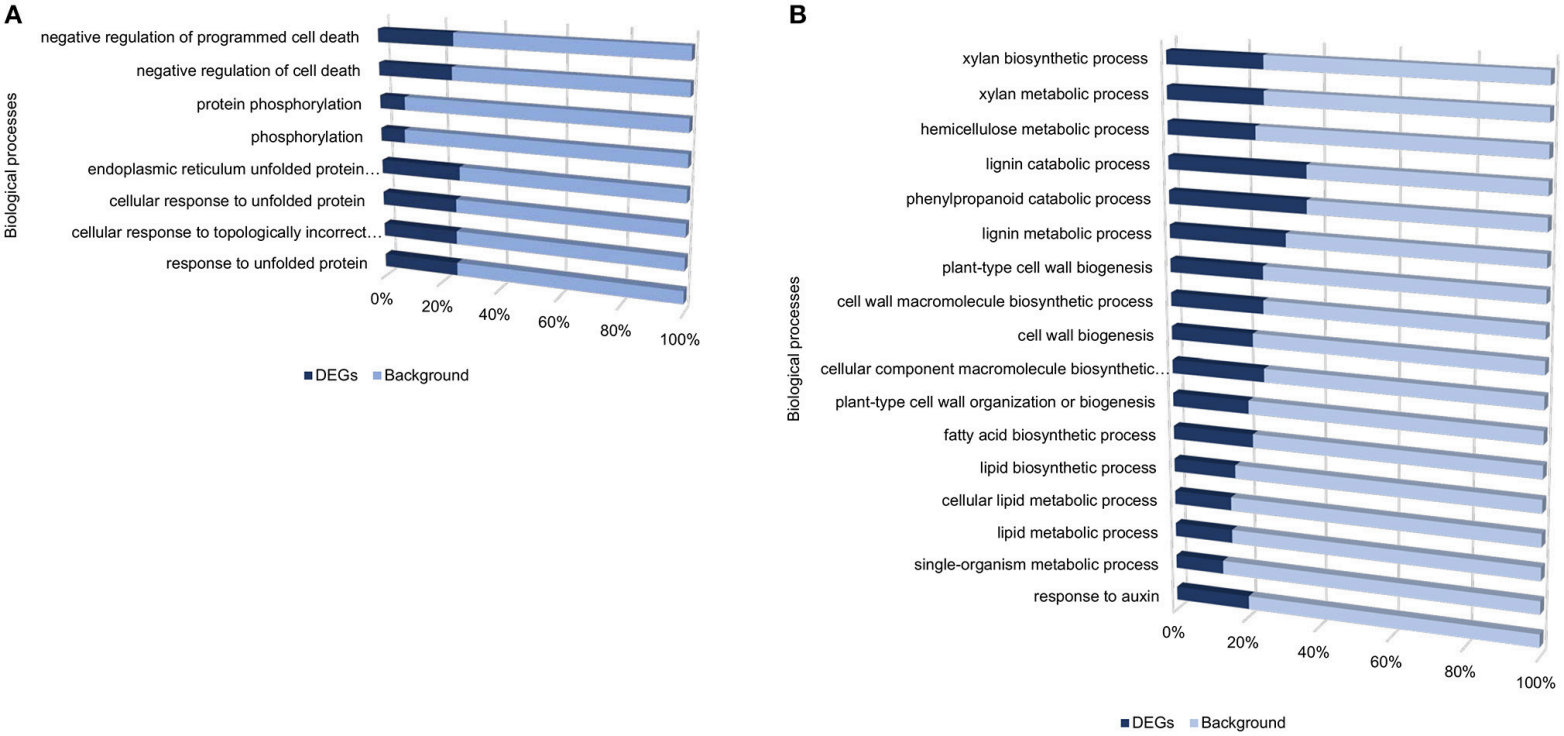
**Gene ontology enrichment analysis showing enriched processes specific to the tolerant cultivar (A)**, and specific to the susceptible cultivar **(B)**, from DEGs identified when comparing inoculated samples to mock-inoculated controls.

**Figure 4:**
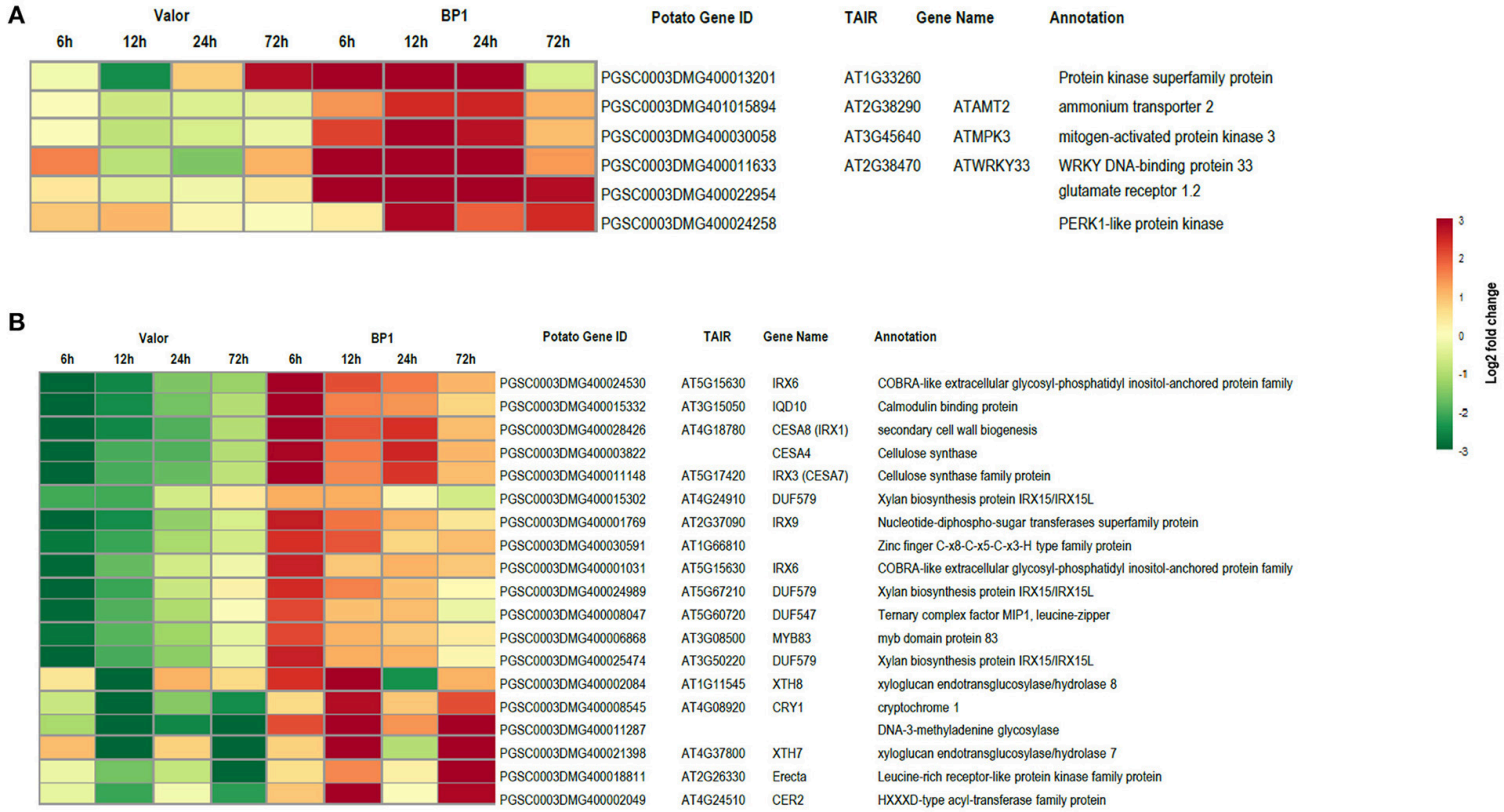
**Heat maps showing transcriptional profiles of selected DEGs from the tolerant and susceptible cultivars. (A)** DEGs important in plant defense responses, up-regulated in the tolerant cultivar. **(B)** Key secondary wall biosynthetic genes down-regulated in the susceptible cultivar.

Despite the high number of DEGs present in both cultivars (4210 DEGs), only 1929 and 4004 DEGs were specific to the tolerant and susceptible cultivars, respectively (Figures 1C,D and Table S5). Among the 1929 DEGs specific to the tolerant cultivar, GO enrichment analyses using g:Profiler webserver revealed that 149 DEGs were overrepresented in the phosphorylation and negative regulation of cell death of GO biological process categories (Figure 3A and Table S6). Interestingly, included in these categories were defense-related signal transduction genes including MPK3 (PGSC0003DMG400030058), key in the activation of plant responses to biotic stress, and MPK4 (PGSC0003DMG401000057) which plays essential roles in pathogen defense signaling (Meng and Zhang, 2013). MPK3 was only induced in the tolerant cultivar in the early stages following infection (at 6, 12, and 24 hpi; ~5.7-Fold) but not in *S. tuberosum* cv Valor (Figure 4A). Plant MAPK cascades are involved in the early transduction of perceived signals from PRRs activating a wide array of downstream defense responses, thus, playing a pivotal role in PTI. Additionally, MPK4 was up-regulated at 12 hpi in cultivar “BP1” (2.3-Fold) but not in “Valor.” Furthermore, defense-related transcription factors (TFs) such as WRKY-like transcription factor (PGSC0003DMG400011633, AtWRKY33) were enriched in this category. PGSC0003DMG400011633 (ortholog of AtWRKY33) was up-regulated in the tolerant cultivar by over 15 fold at 6, 12, and 24 hpi, (Figure 4A). WRKY33 plays key roles in the activation of downstream defense genes. Conversely, DEGs specific to the susceptible cultivar were associated with biological processes such as cell wall biogenesis, regulation of cellular component organization, and cellular response to DNA damage stimulus (Figure 3B and Table S6). Strikingly, genes overrepresented in the “plant-type secondary cell wall biogenesis” comprising mainly secondary wall biosynthetic genes, were mainly down-regulated in the susceptible cultivar. Among these genes were cellulose synthases (e.g., *CESA4, CESA8*, and *FRA8*; PGSC0003DMG400003822, PGSC0003DMG400028426, and PGSC0003DMG400000411 respectively), lignin biosynthesis genes (e.g., *IRX3* and *IRX9*; PGSC0003DMG400011148 and PGSC0003DMG400001769) and NAC domain-containing proteins (e.g., PGSC0003DMG400012113) involved in regulation of secondary wall biosynthesis (Figure 4B and Table S6). Furthermore, MYB83 (PGSC0003DMG400006868, MYB20), which regulates these secondary wall biosynthetic genes, was also down-regulated in the susceptible cultivar (Figure 4B and Table S6). Interestingly, these secondary wall biosynthetic genes were up-regulated in the tolerant cultivar when compared to the susceptible cultivar at each time-point (Figure 4B). Thus, the up-regulation of these genes in the tolerant cultivar following *Pcb1692* infection could imply that they are possibly defense-related genes enhancing resistance to *Pcb1692*.

Furthermore, additional cultivar specific DEGs which potentially contribute to the compatibility or incompatibility of the host-pathogen interaction between potato and *Pcb1692* were also identified in the time-course. Among these were genes up-regulated only in the tolerant cultivar, including genes important in ethylene biosynthesis and signaling pathway such as *ACS4* (PGSC0003DMG400021651, ~19.7), *ACO* homolog (PGSC0003DMG400017190, ~4.3), *EBF1* (PGSC0003DMG400015853, ~2-fold), *ERF1A* (PGSC0003DMG400010750, ~2.9-fold), and *EIL3* (PGSC0003DMG400021381, ~2.3-fold); as well as PGSC0003DMG400016769 (~5.5-fold) and PGSC0003DMG400008337 (MYB21, ~6.7-fold), homologs of WRKY33 and MYB63, respectively, important in regulation of defense responses and secondary cell wall biogenesis, respectively (Table S5). In the susceptible cultivar, genes involved in the ethylene biosynthetic process such as ACC synthases and oxidases; *ACS9* (PGSC0003DMG400021426), *ACO4* (PGSC0003DMG400016714), respectively, were down-regulated (Table S5). Additionally, susceptibility-related genes against necrotrophic pathogens such as MYC2 basic helix-loop-helix-leucine zipper (bHLH) transcriptional factors (e.g., PGSC0003DMG400012237) were differentially expressed in the susceptible cultivar, at 72 hpi (Table S5). Induction of MYC2 tends to enhance susceptibility to necrotrophic pathogens (Glazebrook, 2005).

### Identification and functional characterization of novel genes

**Figure 5:**
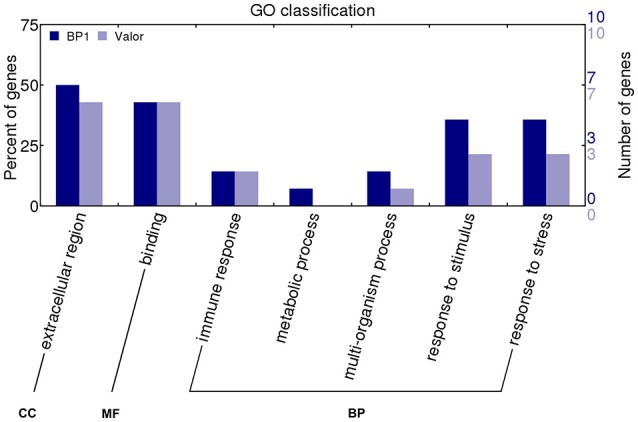
**GO classification of candidate novel CDS genes characterized using InterProScan5**.

In addition to identifying expression profiles of known genes in the potato genome that are induced by *Pcb1692* infection, we also uncovered novel protein-coding potato transcripts responsive to *Pcb1692* inoculation. In a previous study, strand-specific RNA-seq was used to identify a total of 1828 novel CDS gene candidates assembled from reads mapped to intergenic regions using Cufflinks tool (Kwenda et al., 2016). In the present study, these candidate novel transcripts were assessed for their involvement in potato defense responses based on differential expression between cultivars “Valor” and “BP1.” Comparison of *Pcb1692*-inoculated samples to the mock-inoculated controls in each cultivar showed the highest number of DE novel candidate genes was at 12 hpi (549 and 511 in *S. tuberosum* cv Valor and BP1, respectively) (Table S7). Only novel gene candidates showing statistically significant differential expression (adjusted *p*-value < 0.1) and log2 fold change > 1, were considered. Furthermore, by using InterProScan5 (v5.11-51) (Jones et al., 2014), we characterized 28 (including 17 domains) and 32 (including 15 domains) candidate novel transcripts from cultivar “BP1” and “Valor,” respectively (Table S8). The GO terms assigned to each novel CDS transcript were visualized using WEGO (Web Gene Ontology Annotation Plot, http://wego.genomics.org.cn/cgi-bin/wego/index.pl). Candidate novel CDS genes were associated with defense related GO terms, including response to stress and immune response (Novel244, Novel1477, Novel2785, and Novel2787); and response to stimulus (Novel1477, Novel2785, Novel2477, Novel2724, Novel1946, Novel244, and Novel2787) (Figure 5).

**Figure 6:**
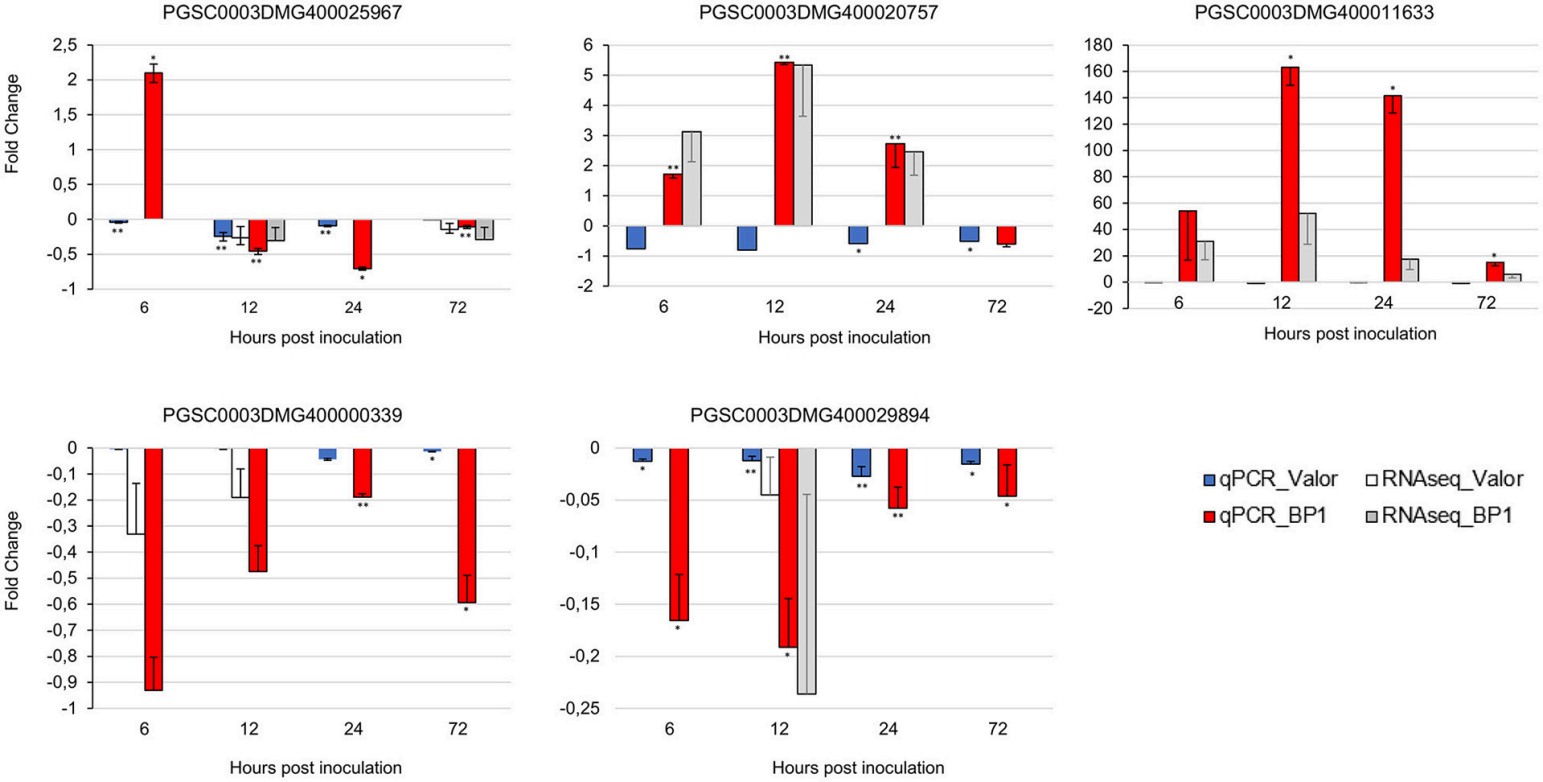
**RT-qPCR validation of RNA-seq gene expression ratios relative to mock inoculated samples using five selected DEGs**. PGSC0003DMG400020757 (Membrane protein), PGSC0003DMG400029894 (Cytochrome P450 hydroxylase), PGSC0003DMG400025967 (Pectinesterase), PGSC0003DMG400000339 (Beta-galactosidase), PGSC0003DMG400011633 (AtWRKY33). Elongation factor 1-α (ef1α) and 18S RNA were used as the reference genes. Error bars represent the range of relative expression (qPCR fold change) calculated by 2^−(ΔΔCt±SD)^ (*n* = 3). The RNA-seq bars at each time-point for each cultivar represent the fold changes calculated from three biological replicates using DESeq2 package, and the error bars represent log2 fold change standard error. Asterisks represent significant differences between inoculated samples and controls determined by Student's *t*-test (^**^P < 0.01; ^*^P < 0.05).

To confirm the time-course RNA-seq data, five DEGs were randomly selected representing genes differentially expressed throughout the time-course in one or both cultivars. The expression profiles of these genes were validated experimentally using RT-qPCR. The RT-qPCR results were in agreement with the RNA-seq expression patterns (Figure 6). In addition, eight of the1828 novel CDS gene candidates were validated using RT-PCR (Figure S1).

## Discussion

To our knowledge, this is the first transcriptome-wide study unraveling responses to soft rot enterobacterial infection in potato stems. Blackleg is an important disease of potato plants mainly in the field. It is caused mainly by members of the genus *Pectobacterium* such as *P. atrosepticum* and the emerging *P. c. brasiliense*. *Pectobacterium* species are usually found in tuber lenticels, on roots or colonizing and occluding potato plant xylem (Charkowski, 2015). Previous work on the host-pathogen interaction between potato and soft rot bacterial pathogens has mainly focused on the pathogen, that is, its pathogenicity and colonization patterns of tubers, roots and stems (Czajkowski et al., 2010; Kubheka et al., 2013). However, not much has been reported on the host potato stem responses against soft rot pathogens. Thus, this study provides new and relevant insights into stem-based defense mechanisms employed by potato plants during colonization and infection of xylem vessels by *P. c. brasiliense*. Therefore, in order to understand potato stem transcriptome dynamics elicited by *P. c. brasiliense* inoculation, we investigated differential gene expression following infection by this pathogen using time-course RNA-seq in tolerant and susceptible potato cultivars. A total of 4718, 4503, 7577, 3505, and 5081 DEGS were identified at 0, 6, 12, 24, and 72 hpi respectively (Figure 1A and Table S3), in pairwise comparisons between cultivars “Valor” and “BP1.” The near exponential increase in up-regulated DEGs in “BP1” induced by *Pcb1692* immediately following inoculation is suggestive of an early activation of defense responses in this tolerant cultivar. Furthermore, the highest number of DEGs was observed at 12 hpi in “BP1” when comparing inoculated samples from each cultivar to mock-inoculated control samples or in pairwise comparisons with “Valor.” This implies that 12 hpi could be a key time-point contributing to the subsequent tolerance in “BP1” (Figures 1A,B). Collectively, these results imply that type of cultivar (in this case “BP1”) has a significant role in defining the early defense responses to bacterial pathogen attack, and these early defenses could lead to overall tolerance or susceptibility, resulting in a compatible or incompatible interaction with *P. c. brasiliense* at the later stages of infection (72 hpi and beyond).

### Pathogen-recognition and signal transduction genes regulated by *Pcb1692* infection

Plants possess pattern recognition receptors (PRRs) which perceive conserved molecular signatures of invading pathogens called PAMPs or recognize signals arising from damage inflicted on the plant by pathogens (DAMPs) in the extracellular environment. Recognition of D/PAMPs initiates plants basal immunity, termed PTI (Dodds and Rathjen, 2010). Generally, PTI defense responses do not involve hypersensitive response (HR) cell death, making PTI important against necrotrophic pathogens. PRRs belong to classes of receptor-like kinases (RLKs). In the present study, several leucine-rich repeats (LRR) RLKs shared between the tolerant and susceptible cultivar were identified (Table 1 and Table S2). Included among these are the well-characterized plant PRRs, such as FLS2, which recognizes the bacterial flagellin conserved peptide (flg22) and homologs of the Arabidopsis EFR receptor which recognizes bacterial EF-Tu (elf18) (Zipfel et al., 2006). Induction EFR genes was much higher in the tolerant cultivar compared to the susceptible cultivar (Table 1). Expression of FLS2, was upregulated in both cultivars up to 12 hpi (Table 1). Perception of bacterial flagellin and EF-TU triggers early defense responses in plants including strong activation of MPK3/MPK6 and MPK4 cascades, ethylene biosynthesis and reactive oxygen species (ROS), which in turn signal downstream defenses such as cell wall strengthening (Meng and Zhang, 2013).

Throughout the time-course WAK receptor genes which perceive DAMPs due to the action of cell wall degrading enzymes, were modulated following inoculation with *Pcb1692* in both cultivars. Some WAKs were up or down-regulated in both cultivars (Table S4). However, most of the WAK1 genes were up-regulated in the tolerant cultivar (e.g., PGSC0003DMG400011792), when comparing inoculated samples from each cultivar to mock-inoculated controls (Table 1). Induction of WAKs has been associated with perception of oligogalacturonides (OGs) and bacterial EF-Tu in defense responses against necrotrophic pathogens (Mengiste, 2012). Thus, the observed up-regulation of WAK1 in BP1 correlates with enhanced pathogen perception. Another crucial RLK is BAK1 which interacts with and forms complexes with PRRs including FLS2, immediately upon perception of D/PAMPs thereby linking the perceived cues with innate immune responses through activation of the MAPK signaling cascades (Mengiste, 2012; Meng and Zhang, 2013). Four genes encoding BAK1 RLKs were differentially expressed in both or one of the cultivars in response to *Pcb1692* infection (Table S4). Three BAK1 encoding genes were up-regulated only in the tolerant cultivar, compared to the susceptible cultivar (Table S5). BAK1 is central in PTI immunity regulation and Arabidopsis *bak1* mutants have higher susceptibility to necrotrophic pathogens (Dodds and Rathjen, 2010). Collectively, these results emphasize the congruence of pathogen recognition in the two cultivars, although, the higher pathogen-induced expression of WAK1 and BAK1 in “BP1” possibly contributes to the strong defense response and observed tolerance in cultivar “BP1.”

Transduction of signals perceived by PRRs and BAK1 complexes is mediated by plant MAPK pathways which transfer signals to downstream components of host immunity. Typically, MAPK cascades comprise MAPK kinase kinase (MAPKKK) which receive signal from PRR/BAK1 complexes. Their activation in turn regulates MAPK kinase (MAPKK) which phosphorylates downstream MAPKs. The MAPK cascades are involved in PTI and ETI and they regulate downstream activities of various substrates including transcription factors (Dodds and Rathjen, 2010). In Arabidopsis flagellin perception can activate two independent MAPK cascade pathways, the MAPKKK-MAPKK (MPKK4/MPKK5)-MPK3/MPK6 cascade and the MAPKKK-MPKK1/MPKK2-MPK4 cascade leading to downstream activation of early defense response genes including WRKY22/29 and WRKY33 transcription (Asai et al., 2002; Meng and Zhang, 2013). In this study, five MAPKKKs (PGSC0003DMG400028666, PGSC0003DMG400018992, PGSC0003DMG400024820, PGSC0003DMG400015448 and PGSC0003DMG400022210) were significantly up-regulated at one or more time-points in one or both cultivars, one MAPKK (, PGSC0003DMG400033696) was up-regulated in “Valor” at 72 hpi. Remarkably, two MAPKs, MPK3 (PGSC0003DMG400030058, AtMPK3) and MPK4 (PGSC0003DMG401000057), critical in flg22-PTI immune responses were only up-regulated in BP1 at 6, 12, and 24 hpi. MPK3 and MPK4 play essential roles in signaling pathogen-induced plant disease resistance, by activation of WRKY33 and WRKY22 in PTI-related defense responses (Asai et al., 2002). In Arabidopsis, MPK4 represses salicylic acid (SA)-dependent resistance (which often result in HR-cell death and are important in defenses against biotrophs) and it interacts with intermediate substrates which activate WRKY33 downstream (Andreasson et al., 2005). Strikingly, WRKY33 was up-regulated only in the tolerant cultivar throughout the time-course in response to *Pcb1692* infection. Taken together, these findings suggest that the early and persistent induction of MPK3/MPK4 cascade genes and the activation of downstream transcription factors in the tolerant cultivar enhance transduction of the perceived stress stimuli leading to stronger cellular defense responses in “BP1” against *P. c. brasiliense* challenge.

### Transcription factors responsive to *P. carotovorum* subsp. *brasiliense* infection

We identified DEGs representing four families of transcription factors (MYB, MYC2 (bHLH), AP2/ERF, and WRKY) modulated in response to *Pcb1692* infection. Timely regulation and coordinated expression of genes in plant immune response signaling pathways is central to effective defense against pathogens (Mengiste, 2012). Transcription factors connect pattern recognition receptors (PPR) perception and MAPK signaling to downstream gene expression. Many families of transcription factors such as ERFs, MYBs, and WRKYs are involved in immunity to necrotrophic pathogens (Lai and Mengiste, 2013). For instance, in this study, WRKY33 associated with plant disease resistance was only up-regulated in the tolerant cultivar in response to *Pcb1692* challenge. WRKY33, a pathogen-inducible transcription factor, was constitutively expressed throughout the time-course (~22.1-fold induction) in the tolerant cultivar. WRKY33 impacts significantly immune responses to necrotrophs. Overall, WRKY33 activates cellular responses downstream of MPK3/MPK6 and MPK4 in PTI immune signaling induced by bacterial flagellin (Asai et al., 2002; Meng and Zhang, 2013).

Defense-related ERFs integrate signals from jasmonate and ethylene pathways in order to transcriptionally activate plant defense responses to necrotrophs (Mengiste, 2012). Here, ERF1 genes were significantly expressed in both cultivars at all the time-points except at 6 hpi in the susceptible cultivar. DEGs for ERF1 were more induced in BP1 compared to Valor in the time-course. ERF1 positively regulates plant resistance to necrotrophs. However, some ERF genes such as ERF4 and ERF5, associated with susceptibility to necroptrophs were also identified and were differentially expressed in the susceptible cultivar following inoculation with *Pcb1692*. The homolog of *AtERF2*, PGSC0003DMG400026261, was induced in both cultivars at 6 hpi but specifically expressed in the tolerant cultivar at 12 and 24 hpi and in the susceptible cultivar at 72 hpi. Interestingly, ERF2 expression patterns showed a 6-fold increase in BP1 at 12 hpi when compared to Valor, indicative of an enhanced and stronger defense response in the tolerant cultivar. In Arabidopsis, *AtERF4* negatively regulates expression of jasmonate responsive defense genes and resistance to necrotrophs. In contrast, *AtERF2* is a positive regulator of defense genes in the jasmonate signaling pathway, conferring resistance to necrotrophic pathogens (Grennan, 2008).

Other differentially expressed transcription factors belong to MYB and MYC families. MYC2 is associated with repression of responses to necrotrophic pathogen infection (Mengiste, 2012) and was mostly down-regulated throughout the time-course in both cultivars, except at the later stages of infection (72 hpi) in the susceptible cultivar when blackleg symptoms are evident. The MYB transcription factor, MYB83 (PGSC0003DMG400006868) was repressed in the susceptible within the time-course, but was up-regulated in the tolerant cultivar (when using pairwise comparisons between cultivars “Valor” and “BP1” at each time-point). MYB83 is a close homolog of and acts redundantly with MYB46. MYB46/MYB83 transcription factors are master regulators of secondary cell wall formation in Arabidopsis, directly regulating expression of genes involved in lignin, cellulose and hemicellulose biosynthesis, including among others, *SND1, CESA4*, CESA7, *CESA8*, MYB58, MYB56, and MYB63 (Mccarthy et al., 2009; Ko et al., 2014). Thus, MYB83 appears to be a key component of cell wall modifications in downstream (late) plant defense responses.

## Conclusion

In this study, we presented the first time-course RNA-seq analysis focusing on potato stem-based defense responses to *P. c. brasiliense* attack. Our findings suggest that differential regulation and expression of PTI-related genes play a central role in cultivar “BP1” pathogen induced defense responses. In addition, by detecting cultivar specific DEGs, we identified gene sets that distinguished the tolerant and susceptible cultivars. Thus, the type of cultivar has a role in plant resistance to *Pectobacterium* infection. Furthermore our time-course data showed induction of defense-related genes at different time-points and stronger expression of majority of these genes in the tolerant cultivar. The highest number of DEGs was identified at 12 hpi in the tolerant cultivar, suggesting that key defense mechanisms are regulated early against *P. c. brasiliense* challenge.

## Author contributions

LM conceived the study. PB contributed to the conception of the study. SK, LM designed the experiments. SK, TM performed the experiments. SK conducted the transcriptome data analysis. SK, LM wrote the manuscript. PB critically reviewed the manuscript. All authors approved the final manuscript.

## Funding

This research was funded by the National Research Foundation (NRF), South Africa through Thuthuka grant number 69362; Research Development Grant for Y-Rated Researchers 93357; Bioinformatics and Functional Genomics (BFG 93685). Additional support was from The Genomics Research Institute, University of Pretoria.

### Conflict of interest statement

The authors declare that the research was conducted in the absence of any commercial or financial relationships that could be construed as a potential conflict of interest.

## Supplementary material

Figure S1 **RT-PCR validation of eight novel CDS candidates using agarose gel**. Lane 1. 1 kb DNA ladder, Lane 2 and 3. Novel1253 in the tolerant and susceptible cultivars, respectively, Lane 4 and 5. Novel917 in the tolerant and susceptible cultivars, respectively, Lane 6, Novel806 in susceptible cultivar, Lane 7 and 9. Novel2142 in the susceptible cultivar, Lane 8. Novel1481 in the tolerant cultivar, Lane 10. Novel750 in the susceptible cultivar, Lane 11. Novel2049 in the susceptible cultivar, Lane 12. Novel2769 in the susceptible cultivar.

Table S1 **Mapping statistics**.

Table S2 **List of identified novel CDS gene candidates**.

Table S3 **List of differentially expressed genes at each time-point between cultivars “Valor” and “BP1**.”

Table S4 **Common DE genes present in both “Valor” and “BP1” at each time-point compared to mock-inoculated samples**.

Table S5 **Cultivar-specific differentially expressed genes**.

Table S6 **Cultivar-specific DEGs overrepresented in various Gene Ontology biological processes**.

Table S7 **Differentially expressed candidate novel CDS transcripts in the time-course**.

Table S8 **Domains assigned to novel CDS candidates using InterProScan5 software tool**.

Table S9 **RT-qPCR primers used to validate RNA-seq data**.

Table S10 **RT-PCR primers used for novel CDS candidate transcripts validation**.

## Abbreviations

CDS: coding DNA sequence
Pcb1692: *Pectobacterium carotovorum* subsp. *brasiliense*
RT-PCR: reverse transcription PCR
RT-qPCR: quantitative reverse transcription PCR
SRE: soft rot Enterobacteriaceae
hpi: hours post inoculation
PCWDEs: plant cell wall degrading enzymes
DEGs: differentially expressed genes.
