# Supplementary figures and images for: RNA-seq Profiling Reveals Defense Responses in a Tolerant Potato Cultivar to Stem Infection by Pectobacterium carotovorum ssp. brasiliense
Source: Front Plant Sci. 2016 Dec 20;7:1905. doi: 10.3389/fpls.2016.01905 (PMC5167753; doi:10.3389/fpls.2016.01905)

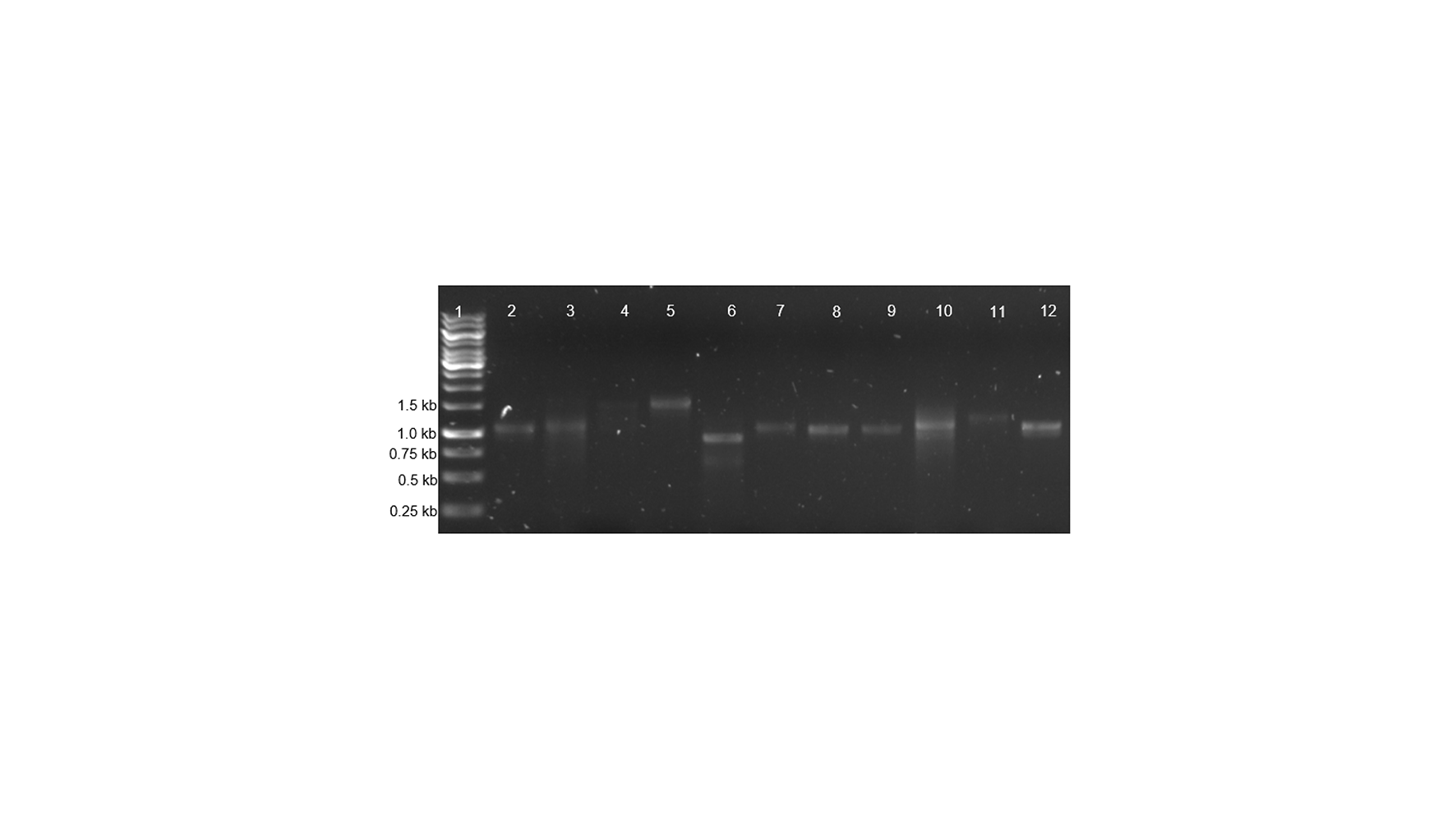

Supplement: Figure S1 — RT-PCR validation of eight novel CDS candidates using agarose gel. Lane 1. 1 kb DNA ladder, Lane 2 and 3. Novel1253 in the tolerant and susceptible cultivars, respectively, Lane 4 and 5. Novel917 in the tolerant and susceptible cultivars, respectively, Lane 6, Novel806 in susceptible cultivar, Lane 7 and 9. Novel2142 in the susceptible cultivar, Lane 8. Novel1481 in the tolerant cultivar, Lane 10. Novel750 in the susceptible cultivar, Lane 11. Novel2049 in the susceptible cultivar, Lane 12. Novel2769 in the susceptible cultivar. [file Image1.TIF]
